# The role of tumor-infiltrating lymphocytes in cholangiocarcinoma

**DOI:** 10.1186/s13046-022-02340-2

**Published:** 2022-04-07

**Authors:** Dong Liu, Lara Rosaline Heij, Zoltan Czigany, Edgar Dahl, Sven Arke Lang, Tom Florian Ulmer, Tom Luedde, Ulf Peter Neumann, Jan Bednarsch

**Affiliations:** 1 Department of Surgery and Transplantation, University Hospital RWTH Aachen, Pauwelsstrasse 30, 52074 Aachen, Germany; 2 Institute of Pathology, University Hospital RWTH Aachen, Aachen, Germany; 3 NUTRIM School of Nutrition and Translational Research in Metabolism, Maastricht University, Maastricht, the Netherlands; 4 Department of Gastroenterology, Hepatology and Infectious Diseases, University Hospital Heinrich Heine University Duesseldorf, Duesseldorf, Germany; 5 Department of Surgery, Maastricht University Medical Center (MUMC), Maastricht, The Netherlands

**Keywords:** Cholangiocarcinoma, Tumor-infiltrating lymphocytes (TIL), Molecular pathogenesis, Oncological prognosis, Immunotherapy, Systematic review

## Abstract

Cholangiocarcinoma (CCA) is the second most common primary liver cancer and associated with a dismal prognosis due to the lack of an efficient systemic therapy. In contrast to other cancers, new immunotherapies have demonstrated unsatisfactory results in clinical trials, underlining the importance of a deeper understanding of the special tumor microenvironment of CCA and the role of immune cells interacting with the tumor. Tumor-infiltrating lymphocytes (TILs) are an important component of the adaptive immune system and the foundation of current immunotherapy. Therefore, the aim of this systemic review is to summarize the current literature focusing on the proportions and distribution, molecular pathogenesis, prognostic significance of TILs and their role in immunotherapy for CCA patients.

In CCA, CD8+ and CD4+ T lymphocytes represent the majority of TILs and are mostly sequestered around the cancer cells. CD20+ B lymphocytes and Natural Killer (NK) cells are less frequent. In contrast, Foxp3+ cells (regulatory T cells, Tregs) are observed to infiltrate into the tumor. In the immune microenvironment of CCA, cancer cells and stromal cells such as TAMs, TANs, MSDCs and CAFs inhibit the immune protection function of TILs by secreting factors like IL-10 and TGF-β. With respect to molecular pathogenesis, the Wnt/-catenin, TGF-signaling routes, aPKC-i/P-Sp1/Snail Signaling, B7-H1/PD-1Pathway and Fas/FasL signaling pathways are connected to the malignant potential and contributed to tumor immune evasion by increasing TIL apoptosis. Distinct subtypes of TILs show different prognostic implications for the long-term outcome in CCA. Although there are occasionally conflicting results, CD8+ and CD4+ T cells, and CD20+ B cells are positively correlated with the oncological prognosis of CCA, while a high number of Tregs is very likely associated with worse overall survival. TILs also play a major role in immunotherapy for CCA.

In summary, the presence of TILs may represent an important marker for the prognosis and a potential target for novel therapy, but more clinical and translational

data is needed to fully unravel the importance of TILs in the treatment of CCA.

## Background

Cholangiocarcinoma (CCA) is a heterogeneous group of cancer originating from the intra- and extrahepatic bile ducts and is considered to be the second most common liver cancer accounting for 10 –15 % of all primary hepatobiliary malignancies [1]. Radical surgical resection or liver transplantation remain the only curative treatments, however, even with a highly radical surgical approach, recurrence rates are reported to be up to 50% [2, 3]. A growing body of research implies that the CCA phenotype is determined not just by genetic and epigenetic alterations in the cancer cells, but also by an extensive molecular crosstalk between those malignant cells and the surrounding tissue microenvironment [4].

Based on the specific immune microenvironment, CCA can be categorized depending on the presence or lack of immune cell infiltration into two groups: cancers that have been infiltrated by lymphocytes and tumors that have not been infiltrated [5]. Immune cell infiltrated tumors are considered immunologically responsive as tumor cells are surrounded by various infiltrating inflammatory cells (e.g. T cells, B cells, myeloid lineage leukocytes, natural killer (NK) cells, macrophages and/or dendritic cells) that contribute to either pro- or anti-tumor activities [6]. Among the invading inflammatory cells, tumor infiltrating lymphocytes (TILs) (e.g. T cells, B cells and NK cells) are the most important determinants of the host immune response against tumor cells. TILs are responsible for the development of anti-tumor immune responses, and may detect tumor antigens and kill tumor cells [7]. Many studies report a survival benefit associated with the presence of TILs in various tumor entities [8]. Tumor cells, on the other hand, may regularly control immunological checkpoints like programmed death-1 (PD-1) and cytotoxic T-lymphocyte antigen-4 (CTLA-4), which are overexpressed on T cells. This creates an immunosuppressive tumor microenvironment and enables an escape to the immune responses. As a result, the restoration of anti-tumor immune response to attack tumoral cells by modern immunotherapy, such as adoptive cell therapy (ACT) and immune checkpoint therapy is becoming increasingly popular [9, 10]. It should be noted that preliminary results of the TOPAZ-1 trial evaluating durvalumab in combination with in advanced CCA displayed encouraging results, revealing that the checkpoint inhibitor durvalumab + gemcitabine & cisplatin (GemCis) significantly improved overall (OS) and progression-free survival (PFS) in patients with advanced CCA compared to placebo + GemCis with acceptable safety margins. This implies that durvalumab + GemCis could be a new first-line standard of care regimen in the near future [11].

In contrast to other cancers as melanoma, renal cell carcinoma, non-small cell lung cancer and adenocarcinoma of the colon, the majority of clinical trials investigating immunotherapy for advanced CCA have demonstrated unsatisfactory outcomes [12, 13]. Compared to other malignancies, the role of TILs, which are the most important actor in the adaptive immunoresponse, therefore, remains to be elucidated in CCA [14]. Based on these, the purpose of this systematic review is to comprehensively summarize the proportions and distribution, molecular pathogenesis, prognostic significance and potential for immunotherapy related to TILs in CCA.

## Methods

### Search strategy

The PRISMA (Preferred Reporting Items for Systematic Reviews and Meta-analyses) criteria were used to conduct this review [15] and this systematic review was registered in the International Prospective Register of Systematic Reviews (PROSPERO) with the ID CRD42021271435. PubMed, Medline, Google Scholar and Web of the Science were searched with the following full-text terms: “T lymphocytes” OR “B lymphocytes” OR “Natural killer (NK) cells” OR “Tumor-infiltrating lymphocytes (TILs)”AND “Cholangiocarcinoma (CCA)” OR “Biliary tree cancers (BTC)” OR “Intrahepatic CCA (iCCA)” OR “Perihilar CCA (pCCA)” OR “Distal CCA (dCCA)”. Boolean operators ‘OR’ was used to combine all expressions of cases including abbreviation while ‘AND’ was used to include lymphocytes in conjunction with CCA in the search. During the literature search, no proximity operators were used. Two authors conducted two independent literature searches both using the same strategy. No additional papers were chosen after the reference list and citation search were completed. No screening for unpublished literature was conducted.

### Include and exclusion criteria

Two authors (DL and JB) screened titles and abstracts for the following criteria: (i) all studies reported on lymphocytes in CCA tissue; (ii) studies were published between 2000 and 2021 and written in English; (iii) publications with available full-text (all identified publications were available to the authors to be included in this review); and (iv) based upon original research. The exclusion criteria were review papers, letters, comments or abstracts;

### Data extraction

The titles and abstracts of all discovered records were independently assessed by two authors after removing the duplicates. Consensus and consultation with a third senior author was used to resolve all differences (UPN). The following data were extracted from included studies: the first author, publication year, country of study, patients’ number, sample size, study type and characteristics, cut-off values of the high/positive rates for TIL expression, length of follow-up, genes analyzed for mutation, anatomic location of tumors, stage at diagnosis, clinical outcomes, and endpoints. Data were organized in standardized tables.

### Risk of bias

The Newcastle-Ottawa scale was used to assess the risk of bias in translational studies reporting oncological outcome [16]. The scale's score range is determined by the study's design. A quality score was derived for case-control studies based on three categories: group selection (four items), comparability across groups (one item), and outcome and exposure evaluation (three items). Each item in the group selection and outcome and exposure evaluation categories received a maximum of one point. Comparability received a maximum of two points. The same three criteria were used in cross-sectional studies to assess the quality. As a result, the maximum score of the scale is nine points with studies being categorized as low (0-3 points), moderate (4-6 points) and high quality (7-9 points), respectively.

## Results

**Fig. 1:**
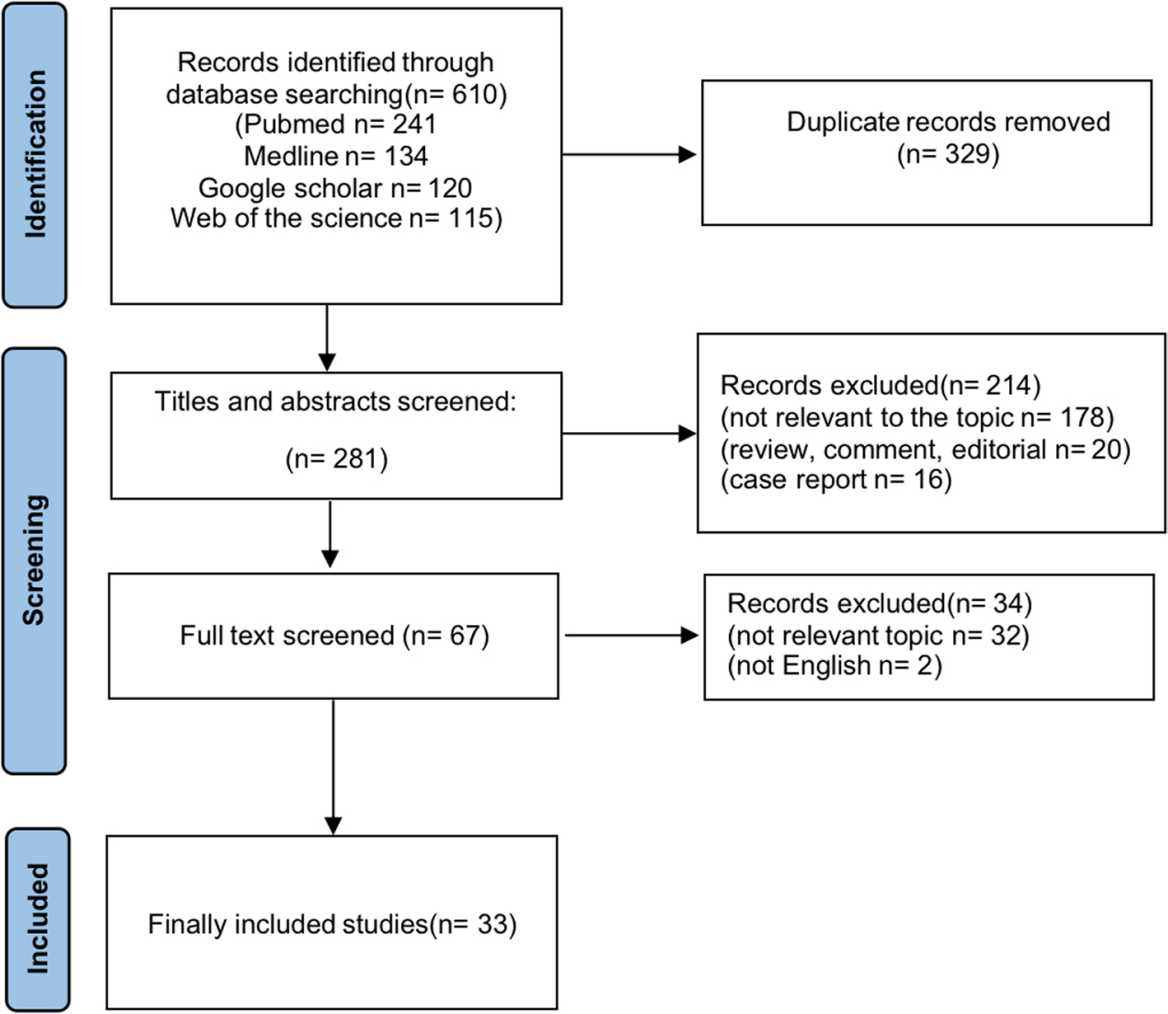
PRISMA flowchart of study selection for this systematic review

A total of 610 records were found in the electronic databases at initial assessment of which 281 titles/abstracts were examined after 329 duplicates have been removed. A total of 178 articles were not associated with the topic and therefore excluded as well as 20 reviews, comments and editorials and 16 case reports. In summary, 67 full text articles were retrieved and reviewed entirely with only 33 of them having met all eligibility requirements. No further studies were found in the reference lists of the included publications and the grey literature (Fig. 1). The quality of studies reporting oncological outcome according to the Newcastle-Ottawa scale is presented in Table 1.

**Table 1:** Quality assessment of included clinical studies

Ref	Author	Selection	Comparability	Outcomes	Quality score
[17]	Asahi Y	★★★★	★	★	6/9
[18]	Goeppert B	★★★★	★	★★	7/9
[19]	Hasita H	★★★★	★★	★★	8/9
[20]	Kim HD	★★★	★★	★	6/9
[21]	Kim R	★★★	★★	★★	7/9
[22]	Kitano Y	★★★★	★★	★★	8/9
[23]	Lu JC	★★★★	★★	★	7/9
[24]	Miura T	★★★★	★★	★★	9/9
[25]	Oshikiri T	★★★★	★★	★	7/9
[26]	Tian L	★★★★	★★	★★★	9/9
[27]	Ueno T	★★★★	★★	★★★	9/9
[28]	Vigano L	★★★	★★	★★	7/9
[29]	Wu H	★★★★	★★	★★	8/9
[30]	Xu YP	★★★★	★★	★★	8/9

All included translational studies reporting oncological outcome were evaluated in accordance with the Newcastle-Ottawa scale [16]. The maximum score of the scale is nine points with studies being categorized as low (0-3 points), moderate (4-6 points) and high quality (7-9 points), respectively

### Investigative methods

To investigate TILs and other cells of the microenvironment in CCA, previous studies have used immunohistochemistry (IHC) including conventional H&E staining and multiplexed immunohistochemistry to explore the distribution and characterisation of TILs (Tables 2 and 4). Pathological and immunohistochemical examinations were usually performed by two or more observers who were blinded on the clinical data. It should be noted that there is a large heterogeneity in terms of the defined cut-offs used by previous studies. Some studies use percentiles, tertials or the median, whereas others use absence vs presence, or do not report a cutoff at all. Molecular studies in reference to TILs in CCA frequently utilized flow cytometry-based techniques (Table 3).

**Table 2:** Characteristic distribution of TILs in CCA

Ref	Author	Year	Country	Sample(n)	Location of TILs	Subtypes of TILs	Assessment of TILs	Distribution (number or density of TILs)
[31]	Zhou G	2019	China	CCA (26)	IT vs. PT	CD8+ / FoxP3+ / CD4+ / CD56_+_	IHC	CD8+, CD4+: PT > IT; Foxp3+, CD56: no difference
[17]	Asahi Y	2020	Japan	iCCA (78)	IT vs. PT	CD8+ / FoxP3+	IHC	CD8+: PT > IT (91.0±89.9 vs. 41.1±54.1) Foxp3+: PT > IT (18.9±21.5 vs. 11.5±15.7)
[23]	Lu JC	2019	China	iCCA (320)	IT vs. PT	PD1(+)T	IHC	PT < IT (40±5 VS 60.1±6.5; *p*< 0.01)
[32]	Kasper HU	2009	China	CCA (27)	IT vs. PT	CD3+ / CD4+ / CD8+/CD20	IHC	PT > IT CD3: 52.6±28.5 vs 310.4± 202.0, *p*=0.008 CD4: 18.0±22.3 vs 223.1±43.2, *p*=0.043 CD8: 40.7± 30.5 vs 118.7± 35.5, p≤ 0.001 CD20: 11.1 (± 11.8) vs 0.1 (± 0.3), *P* = 0.035
[27]	Ueno T	2018	Japan	eCCA (117)	IT vs. PT	CD4+ / CD8+ / FoxP3+	IHC	No difference CD4+(median 77 vs 59, *p*=0.16) CD8+(median 52 vs 55, *p*=0.94) Foxp3+(median 9 vs 9, *p*=0.62)
[18]	Goeppert B	2013	Germany	eCCA (149) iCCA (157) GBAC (69)	IT vs. PT	CD4+ / CD8+ / FoxP3+/CD20	IHC	CD4+: PT > IT CD8+: PT > IT CD20: No foud Foxp3+: PT < IT
[30]	Xu YP	2021	China	iCCA (140)	IT vs. PT	CD8+	IHC	PT > IT
[26]	Tian L	2020	China	iCCA (322)	IT vs. PT	CD8+	mIHC	PT > IT (*p*<0.001)
[29]	Wu H	2021	China	iCCA (50)	IT vs. PT	CD8+ / CD3+	IHC	PT > IT (*p*=0.009, *p*=0.047)
[20]	Kim HD	2021	Korea	CCA (52)	IT vs. PT	CD8+ / CD4+ / FoxP3+	mIHC	PT > IT (*p*<0.001, *p*<0.001, *p*<0.001)
[33]	Kim HD	2021	Korea	iCCA (33)	IT vs. PT	CD103+CD8+	mIHC	PT < IT (mean 1.4/mm^2^ vs 1.8/mm^2^)

Various studies investigated the spatial distribution of tumor-infiltrating lymphocytes in cholangiocarcinoma

*CD*, cluster of differentiation, *CCA* cholangiocarcinoma, *eCCA* extrahepatic cholangiocarcinoma, *FOXP3* forkhead box P3, *GBAC* gallbladder cancer, *iCCA* intrahepatic cholangiocarcinoma, *IHC* Immunohistochemistry, *IT* intratumoral, *mIHC* multiplexed immunohistochemistry, *PD* programmed cell death protein, *PT* peritumoral, *Ref* reference, *Th* helper T cell, *TIL* tumor-infiltrating lymphocytes

**Table 3:** Molecular pathogenesis of CCA related to TILs

Ref	Author	Year	Country	Investigated genes, cytokines or molecular pathways	Sample size	Tumor type	Experimental materials	Experimental methods and assessment of TILS	Main findings
[34]	Yoon JG	2021	Korea	KRAS and CIN	121	iCCA=33, eCCA=35, dCCA=53	tissue	targeted sequencing, IHC	KARS altered and chromosomal instable tumors are associated with resistance to immunotherapy.
[33]	Kim HD	2021	Korea	Wnt/β-catenin and TGF-β signalling pathways	33	iCCA	blood, tissue	flow cytometry, multiplexed IHC, RNA sequencing	Wnt/β-catenin and TGF-β signalling pathways decrease CD69+CD103+ TRM-like CD8+ TILs.
[35]	Fukuda Y	2020	Japan	CXCL9	70	iCCA	tissue	IHC	In iCCA, CXCL9 expression is closely correlated with prolonged postoperative survival and an increased number tumor-infiltrating NK cells.
[36]	Goeppert B	2019	Germany	Microsatellite instability (MSI-H)	308	iCCA=159,pCCA=106,dCCA=43	tissue	TMA, IHC, DNA extraction	MSI-H CCA is associated with a high number of TILs.
[37]	Cornillet M	2019	Sweden	KIR and HLA gene	148	CCA	blood	rtPCR, IHC, flow cytometry, RNA, sequencing	HLA and KIR-positive NK cells infiltrate CCA.
[38]	Thepmalee C	2018	Thailand	IL-10 and TGF-b	n. a.	n. a.	cell	cell culture, Western blot, IHC	Inhibition of IL-10 and TGF-b enhances T-cell response against CCA cells.
[39]	Panya A	2018	Thailand	PRKAR1A	n. a.	n. a.	tissue, cell	IHC, DNA sequencing, Western blot, ELISA	Activated T cells are significantly associated with the expression levels of PRKR1A in CCA cells.
[40]	Qian Y	2017	China	aPKC-i/P-Sp1/Snail Signaling	64	CCA	tissue, cell	IHC, Cell culture, Western blot, PCR, quantitative real-time flow cytometry	aPKC-i/P-Sp1/Snail signaling may play an important role in recruiting TILs.
[41]	Junking M	2017	Thailand	RNA-pulsed dendritic cells	n. a.	CCA	cell	cell culture, flow cytometry	Pooled mRNA from three CCA cell lines significantly increased the specific killing capacity of activated T lymphocytes.
[42]	Carnevale G	2017	Italy	Fas/FasL pathway	n. a.	iCCA	cell	cell culture, Western blot, IHC, flow cytometry	iCCA cells have immune-modulatory properties by inducing apoptosis of T and NK cells via the Fas/FasL pathway.
[43]	Duan SG	2010	China	MAPK-ERK pathway	n. a.	CCA	cell	cell culture, Western blot, real-time PCR, animal model, RNA isolation and silencing	Laminin-mediated MAPK-ERK pathway induces FasL Expression, subsequently CCA cells kill the Fas-expressing TILs.
[44]	Ye Y	2009	China	B7-H1/PD-1 pathway	31	iCCA	tissue	IHC, DNA extraction	B7-H1/PD-1 pathway may be linked to malignant potential of iCCA and contribute to tumor immune evasion by promoting CD8+TILs apoptosis.

Various studies investigated the molecular pathogenesis related to tumor-infiltrating lymphocytes in cholangiocarcinoma

*aPKC* atypical protein kinase C-iota, *CCA* cholangiocarcinoma, *CIN* chromosomal instability, *CXCL9* Chemokine (C-X-C motif) ligand 9, *dCCA* distal cholangiocarcinoma, *ELISA* Enzyme-Linked ImmunoSorbent Assay, *HLA* Human leukocyte antigen, *iCCA* intrahepatic cholangiocarcinoma, *IHC* immunohistochemistry, *KIR* Killer cell immunoglobulin-like receptor, *MAKP-ERK* Mitogen-activated protein kinases-Extracellular signal-regulated kinases, *mRNA* messenger RNA, *MSI-H* Microsatellite instability, *NK* natural killer, *pCCA* perihilar cholangiocarcinoma, *PCR* Polymerase chain reaction, *PRKAR1A* protein kinase CAMP-dependent type I regulatory subunit alpha, *Ref* reference, *TGF* transforming growth factor, *TMA* tissue microarray analysis, *TRM* tissue-resident memory

### Proportions and distribution of TILs in CCA

In comparison to hepatocellular carcinoma (HCC), CCA has a lower number of CD8+ T cells in total, but concentrated regulatory T cells (Tregs) and a higher level of immunoinhibitory checkpoints [32]. When compared to the healthy liver, tumors displaye lower proportions of cytotoxic T cells and NK cells, but higher proportions of Tregs [31]. The most common type of inflammatory cells were T lymphocytes. CD8+ T lymphocytes made up most of the T lymphocytes, whereas CD4+ T lymphocytes were also common. B lymphocytes were only seen occasionally. The total number of NK cells was also modest, though higher than of B cells [18].

In 11 of the 33 studies, the distribution of TILs between peritumoral and intratumoral areas in CCA was studied [17, 18, 20, 23, 26, 27, 29–33] (Table 2). For iCCA, 5 studies show that CD8+, CD4+ and CD3+ T cells mainly distributed around the cancer itself [17, 18, 26, 29, 30]. One study observed Foxp3+ T cells directly infiltrating into the tumor [18], while another study could not support these results [17]. Interestingly, CD8+ T cells combining special molecular factors like PD1 (+) or CD103 mainly distributed in the cancer core in iCCA [23, 33]. For eCCA, one study showed that CD8+ and CD4+T cells are mainly located in peritumoral and Foxp3+ T cells in the intratumoral areas [18] and another publication found no difference between intratumoral and peritumoral areas for CD8+, CD4+T and Foxp3+ T cells [27]. For CCA (including both iCCA and eCCA), three studies revealed that CD8+, CD4+ and CD3+ T cells mainly infiltrated outside of the tumor [26, 31, 32]. Foxp3+ T cells were also found located in peritumoral areas mostly [20]. However, another study by Zhou et al. did not observe any significant difference for the location of Foxp3+ T cells [31]. In contrast to T cells, B cells have been poorly examined in CCA and less evidence is available. In one particular study, Kasper et al. showed that CD20+ cells infiltrate more in the peritumoral than in the intratumoral area [32].

**Fig. 2:**
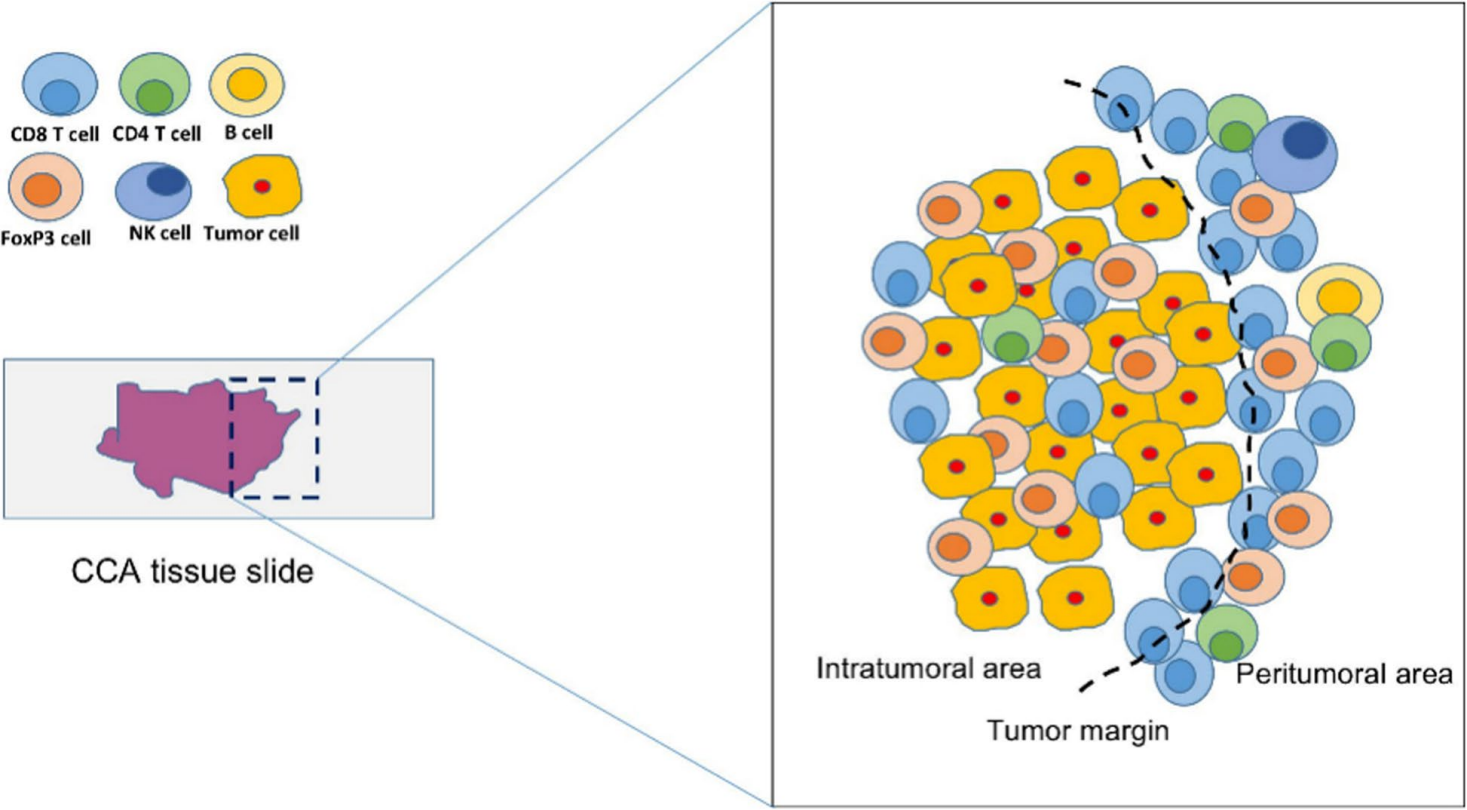
Spatial distribution of tumor-infiltrating lymphocytes in CCA. In CCA, CD8+ T lymphocytes represent the majority of T lymphocytes, whereas CD4+ T lymphocytes were also common. B lymphocytes are only seen occasionally. The total number of NK cells is also modest, though higher than B cells. While CD8+ and CD4+ cells are mainly distributed around the cancer, while Foxp3 cells infiltrate into the tumor. CCA, cholangiocarcinoma; CD; cluster of differentiation; Foxp3, forkhead box p3; NK, natural killer; TGF, transforming growth factor

As suggested by the current literature, it is assumable that CD8+, CD4+ and CD3+ T cells were mainly located in the peritumorual area irrespective of the CCA subtype while especially for Foxp3+ T cell and B cells, further targeted studies are needed to explore their specific location under different situations. An overview of the different cell subsets and their spatial distribution is presented in Fig. 2 and Table 2.

### Molecular Pathogenesis of CCA related to TILs

**Fig. 3:**
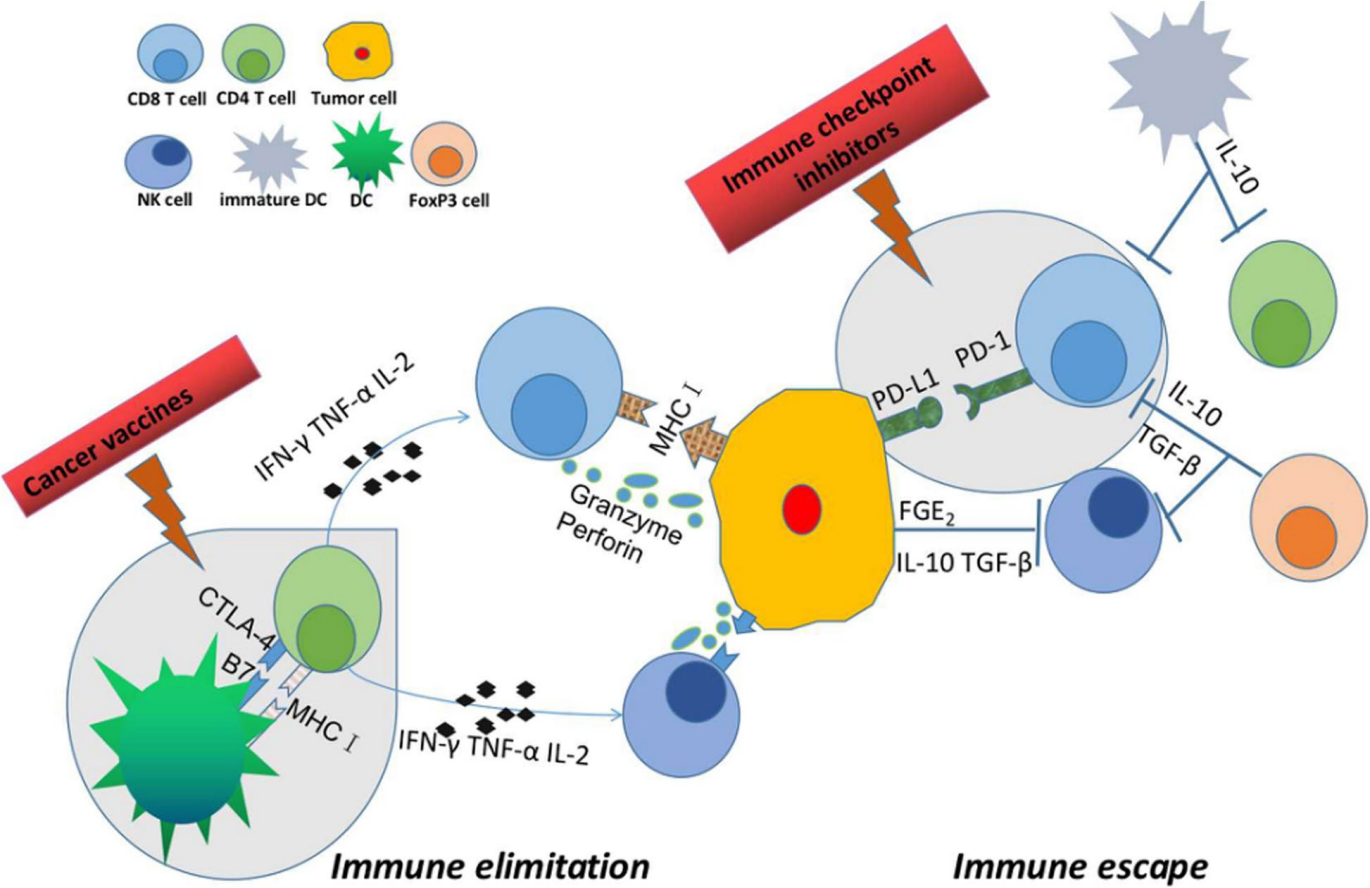
Overview of different types of tumor-infiltrating lymphocytes in CCA. TILs are a highly heterogeneous group of lymphocytes. Distinct cell subsets play different roles in the tumor microenvironment. CD4+ cells are activated by reaction with peptide antigens delivered by major histocompatibility complex II (MHC II) and secrete cytokines such as IFN-γ, TNF-α and IL-2, which mediate cellular immunity and enhance the killing ability of NK cells and cytotoxic T cells. CD8+ cytotoxic T cells destroy tumor cells directly by releasing chemicals like perforin and granzyme and indirectly by inducing apoptosis by expressing FasL or secreting TNF-α attaching to target cell surface receptors. NK cells kill tumor cells by the same mechanisms as CD8+ cytotoxic T cells. Tregs suppress CD8+ cytotoxic T cells and NK cells by secreting soluble anti-inflammatory chemicals such as IL-10 and TGF-β. CD, cluster of differenciation; FasL, Fas ligand; IFN; interferon; IL, interleukin; MHC; major histocompatibility complex; NK, natural killer; TGF, transforming growth factor; TILs, tumor-infiltrating lymphocytes; TNF, tumor necrosis factor; Tregs; regulatory T cells

TILs are a highly heterogeneous group of lymphocytes and act as key players in important pathways (Fig. 3). RNA sequencing, Western blot, PCR, IHC and other methods were used to investigate the link between various signal pathways and TILs [33, 40, 42–44] (Table 3). Kim et al. found that signature genes of the wingless and Int-1(Wnt)/-catenin and transforming growth factor (TGF)-signaling pathways to be elevated in tumors with low numbers of CD69+CD103+ tissue-resident memory-like CD8+ TIL, which represent prominent tumor-specific immune response and hold promise as a potential therapeutic target in iCCA patients [33]. The atypical protein kinase C-iota (aPKC-i) / Ser59-phosphorylated specificity protein 1 (P-Sp1) / Snail signaling induced immunosuppression by producing immunosuppressive natural T regulatory–like CD4+CD25- cells in 64 CCA patients [40]. Furthermore, Carnevale et al. showed that iCCA cells have the immune-modulatory capability of inducing apoptosis of T and NK cells via the Fas/FasL pathway and avoid inflammatory responses by up-regulating the cellular FADD-like IL-1β-converting enzyme-inhibitory protein (c-FLIP) system [42]. A Chinese group conducted an experiment in which cells were examined after being treated with laminin or transfected with plasmids containing siRNA targeted to the 67-kDa laminin receptor, and observed the induction of FasL expression and cytotoxicity in CCA cells via the mitogen-activated protein kinases - extracellular signal-regulated kinases (MAKP-ERK) pathway against Fas-sensitive Jurkat T cells [43]. Another Chinese study discovered that the B7-H1/PD-1 pathway is linked to the malignant potential of iCCA and contributes to tumor immune evasion by boosting CD8+ TIL apoptosis [44].

The effects of gene mutations on TILs has also been investigated previously [34, 37] (Table 3). In a multidimensional analysis of DNA from 112 blood samples of European patients with CCA, Cornillet et al. found multiple alterations at the killer cell immunoglobulin-like receptor (KIR) and human leucocyte antigen (HLA) gene loci and speculated that these alterations might affect NK cell tumor surveillance [37]. By molecular characterization analysis of 121 CCA patients, a Korean study demonstrated that KRAS mutations with low TIL density in tumors were linked to low immunogenicity in the tumors [34].

Another area of research has been the influence of cytokines, proteins, and nucleic acids in TILs [39, 36, 38, 41, 35] (Table 3). Fukuda et al. investigated that high C-X-C motif ligand 9 (CXCL9) expression was closely correlated with prolonged postoperative survival and an increased number of tumor-infiltrating NK cells. Mice experiment showed that CXCL9 could enhance NK cell recruitment into tumors to conduct anti–tumor immunity [35]. Panya el at. found that the killing ability of effector T cells was associated with intracellular protein kinase CAMP-dependent type I regulatory subunit alpha (PRKAR1A) levels [39]. By conducting cell experiments, Junking et al. revealed that RNA-pulsed dendritic cells could promote the activation anti-tumor effector T-cells against CCA cells. In addition, pulsing dendritic cells with pooled messenger RNA from multiple cell lines enhanced the efficacy of a cellular immune response against CCA cells [39]. DNA mismatch repair deficiency is a major molecular pathway of genetic instability in cancer. Goeppert el at. investigated a cohort comprising 308 CCAs discovering that patients affected by high-level microsatellite instability (MSI-H) had mostly an atypical histomorphology, showed a longer overall survival and higher numbers of CD8 + T cells, FOXP3 + regulatory T cells, CD20 + B cells [36]. Another group discovered that by inhibiting the IL-10 and TGF-b receptors on DCs, the level of IFN-γ and the cytolytic activity of effector T cells on CCA cell lines can significantly be increased. Thus, inhibition of the IL-10 and TGF-b receptors on DCs is crucial in the preparation of DC-activated effector T cells for adoptive T-cell therapy [38].

### Prognostic significance of TILs in CCA

Fourteen of the 33 publications investigated the prognostic significance of TILs in patients with CCA (Tables 1 and 4). For iCCA, 7 studies showed that patients with a high number or density of CD8+ T cells displayed longer OS or DFS [17, 18, 23, 26, 28–30]. Among them, 5 studies investigated the effect of CD8+ T cells with respect to spatial distribution (intratumoral (IT), peritumoral (PT) or tumor margin (TM)) on the prognosis of iCCA and all revealed that a higher number of CD8+ T cells in the tumor margin is associated with prolonged OS. Similarly, a high density of CD4+ T cells in the tumor margin was independently associated with favourable OS or DFS [18, 20]. Four groups investigated the relationship between Foxp3+T cells and prognosis. While Foxp3+T infiltration was associated with shorter OS in two reports [17, 28], one study found a positive prognostic impact [18] and another no significant relationship [19]. Of note, only one study investigated the expression of B cells and observed that the presence of CD20+ cells was associated with an improved prognosis [18].

**Table 4:** The relationship between the number or density of TILs with the prognosis of CCA

Ref	Author	Year	Country	Sample(n)	Location of TILs	Subtype of TILs	Assessment Of TILs	Criteria for cut-off (Positive/High Expression)	Follow-up (months)	Endpoint	Prognostic significance
[17]	Asahi Y	2020	Japan	iCCA (78)	IT / PT	CD8+ / Foxp3+	IHC	Mean (cell count, ×400 HPF)	Not reported	OS	Only a high CD8+ count in the PT area are associated with better OS (*p*=0.0103), other subtypes and locations are not associated with prognosis.
[23]	Lu JC	2019	China	iCCA (320)	IT / PT	PD1(+)T	IHC	Score>3 (5%–9% of the tissue section)	Not reported	OS / RRS	High PD1(+) T cells in ICC patients with HBV infection are associated with inferior OS (*p* =0.026) and a higher RRS (*p*=0.011).
[27]	Ueno T	2018	Japan	eCCA (117)	IT	CD4+ / CD8+ / Foxp3+	IHC	Median (cell count, ×400 HPF)	27 (median)	OS	High numbers of CD4+ TILs are related to better OS (*p*=0.049).
[22]	Kitano Y	2018	Japan	eCCA (114)	IT	CD8+ / Foxp3+	IHC	Mean (cell count, ×400 HPF)	62.6 (median)	OS	Low CD8 count and high number of Tregs are associated with worse OS (*p*=0.04, 0.02).
[21]	Kim R	2018	USA	eCCA (44)	IT	CD8+CD45RO+	IHC	≥100 (cell counts, ×400 HPF)	Not reported	OS	Individuals with CD8+CD45RO+ TILs displayed a longer OS (*p*=0.013).
[28]	Vigano L	2019	Italy	iCCA (43)	IT	CD3+ / CD8+ / Foxp3+	IHC	>0.10% (rate of positive cell staining, 400 HPF)	42 (median)	OS / RFS	CD3+ and CD8+ infiltrate is associated with longer OS (*p*=0.001, 0.051) and lower RRS (*p*=0.01, *p*=0.004), while Foxp3+ infiltrate is associated with shorter OS (*p*=0.049).
[18]	Goeppert B	2013	Germany	eCCA (149) iCCA (157) GBCA (69)	IT / PT	CD4+ / CD8+ / Foxp3+ / CD20+	IHC	median (cell count, ×400 HPF)	Not reported	OS	Patients with intraepithelial tumor-infiltrating CD4+ and CD8+, show a significantly longer OS (*p*=0.002, *p*=0.015). Presence of CD20+ and Foxp3+ T lymphocytes also has a positive prognostic impact (*p*=0.032, *p*=0.018).
[25]	Oshikiri T	2003	Japan	eCCA (58)	IT	CD8+	IHC	>0 (positive cell count, x200 HPF)	Not reported	OS	Intratumoral CD8+T cells are associated with increased OS (*p* =0.0001).
[24]	Miura T	2017	Japan	iCCA (115)	IT	CD8+	IHC	median (cell count, ×400 HPF)	38 (median)	OS	CD8+T cells in cancer cell nests is not related to OS (*p*=0.365).
[19]	Hasita H	2010	Japan	iCCA (55)	IT	Foxp3+	IHC	median (cell count, ×400 HPF)	39.7 (mean)	OS / DFS	Count of Foxp3+ is not correlated with DFS and OS (*p*=0.09, *p*=0.23).
[30]	Xu YP	2021	China	iCCA (140)	IT	CD8+	IHC	Median (cell count, x400 HPF)	25 (median)	OS / DFS	High count of infiltrating CD8+ cells is related to longer OS and DFS.
[26]	Tian L	2020	China	iCCA (322)	IT	CD8+	mIHC	Median (cell count, x400 HPF)	27 (median)	OS / TTR	Patients with a high density of CD8+ T cells displayed longer OS (*p*=0.006) and more favorable TTR (*p*=0.003)
[29]	Wu H	2021	China	iCCA (50)	IT / TM	CD8+	IHC	Median (cell count, x200 HPF)	26.5 (median)	OS / RPF	A higher number of CD8+ cells in the tumor margin is associated with prolonged OS and RFS (*p*=0.048, *p*=0.033).
[20]	Kim H	2021	Korea	CCA (52)	IT / TM	FoxP3-CD4+	mIHC	Median (cell count, x400 HPF)	Not reported	OS / RFS	A high density of FoxP3-CD4+ cells in the tumor margin is independently associated with favorable DFS and OS (*p*=0.005, *p*=0.004).

Various studies reported oncological outcome with respect to tumor-infiltrating lymphocytes in cholangiocarcinoma

*CCA* cholangiocarcinoma, *DFS* disease free survival, *eCCA* extrahepatic cholangiocarcinoma, *FOXP3* forkhead box P3, *GBAC* gallbladder cancer, *HPF* high power field, *iCCA* intrahepatic cholangiocarcinoma, *IT* intratumoral, *PT* peritumoral, *Ref* reference, *RFS* relapse-free survival, *RRS* recurrence rate, *TIL* tumor-infiltrating lymphocytes, *TM* tumor margin

For eCCA, Goeppert et al., Oshikiri et al. and Kitano et al. concluded that a high number of CD8+ T cells translates to better OS or DFS [18, 22, 25] and CD4+ T cells were also found to have a favourable impact by Goeppert et al. and Ueno et al. [18, 27], while Kitano et al. observed that Foxp3+T cells were associated with a dismal prognosis [22].

In summary, CD8+ and CD4+ T cells were mainly positively correlated with the overall prognosis in CCA irrespective of their respective spatial distribution. However, the relationship between Foxp3+T cells and long-term results of CCA remains ill-defined and requires further research. Due to the limited number of studies, the overall prognostic role of B cells is not conclusive and more related research is needed to unravel their defined impact on long-term outcome.

### Potential TILs-related immunotherapy for CCA

A total of 6 of the 33 papers investigated possible TIL-related immunotherapy studies [35–49] (Table 5). CCA cell [46–48] or rodent models [35, 45, 49] were used to investigate cell proliferation, tumor growth and prognosis. Diggs et al. treated iCCA mice with a combination of anti-CD40 and anti-PD-1, resulting in a much lower tumor burden as well as enhanced numbers and activation of CD4+ and CD8+ T cells, NK cells, and myeloid cells in the tumor [45]. Pan et al. discovered that CTLA4–PD-L1 DNA immunization induced the development of specific antibodies and inhibited tumor growth in iCCA rats [49]. In an in-vitro investigation using gemcitabine paired with cytotoxic T-lymphocytes (CTLs) to treat gemcitabine-resistant CCA cells, Sawasdee et al. discovered that gemcitabine increases the cytotoxic activity of effector T cells against chemo-resistant CCA cells [46]. T cells stimulated with Dendritic cells (DC) pulsed with cell lysates of honokiol-treated cancer cells boosted specific killing of human CCA cells substantially more than those stimulated with DCs pulsed with cell lysates of untreated CCA cells [47]. When Morisaki et al. cultured cytokine-activated killing (CAK) cells with cetuximab, an epidermal growth factor receptor antagonist, they found enhanced CAK cells cytotoxicity. Cetuximab may therefore potentially be used to enhance CAK cell therapeutic activity in patients with CCA [48]. CXCL9, an IFN-γ inducible chemokine, has been reported to play versatile roles in the tumor-host relationship. Fukuda et al. found that CXCL9 was released in response to inflammatory stimuli in cholangiocarcinoma cell lines and that CXCL9 did not promote cell growth or cell invasion in CXCL9-expressing cholangiocarcinoma cell lines. In addition, mice treated by silencing CXCL9 with short hairpin RNA got greater tumor burden by disrupting natural killer cell recruitment into tumors. However, Fukuda et al. also revealed that high endogenous CXCL9 expression was correlated with favorable postoperative survival [35].

**Table 5:** Potential TILs-related immunotherapy for CCA

Ref	Author	Year	Country	Experimental methods	Tumor type	Treatment	Outcomes
[45]	Diggs L	2020	USA	Animal model	iCCA	Combined anti-CD40/PD-1	Impaired iCCA cell growth, prolonged mice survival.
[46]	Sawasdee N	2020	Thailand	Cell culture experiment	CCA	Gemcitabine combined with cytotoxic T-lymphocytes (CTLs)	Gemcitabine in combination with CTLs promotes cancer cell death.
[47]	Jiraviriyakul A	2019	Thailand	Cell culture experiment	CCA	Honokiol plus dendritic cells (DC)-based vaccine	T lymphocytes stimulated with DCs pulsed with cell lysates of honokiol-treated CCA cells significantly increased specific killing of human CCA cells compared to DCs pulsed with cell lysates of untreated CCA cells.
[48]	Morisaki T	2012	Japan	Cell culture experiment	CCA	Cytokine-activated killer (CAK) cells with cetuximab	Combining CAK cells with cetuximab significantly enhanced cytotoxicity.
[49]	Pan YR	2020	China	Animal model	iCCA	DNA vaccination targeting CTLA4–PD-L1	DNA vaccination targeting CTLA4–PD-L1 triggered the production of specific antibodies and suppressed tumor growth in an iCCA rodent model.
[35]	Fukuda Y	2020	Japan	Animal model	iCCA	CXCL9	CXCL9 knockout leads to greater tumor burden by disrupting natural killer cell recruitment into the tumor in mice

Various studies investigated potential immunotherapy based on tumor infiltrating lymphocytes in animal models or cell experiments

*CAK* Cytokine-activated killer, *CCA* cholangiocarcinoma, *CTLs* cytotoxic T-lymphocytes, *CTLA4* cytotoxic T-Lymphocyte associated protein 4, *DCs* dendritic cells, *iCCA* intrahepatic cholangiocarcinoma, *PD1* programmed cell death protein 1, *PD-L1* programmed cell death 1 ligand 1, *RFS* relapse-free survival

## Discussion

TILs are present in many solid tumors and form a highly heterogeneous population [50], which mostly include B lymphocytes, CD8+ cytotoxic T lymphocytes, CD4+ T lymphocytes, and FoxP3+ Tregs. Spatial heterogeneity is one of the key features of the tumor microenvironment [51] and the composition and localization of immune infiltrate substantially varies depending on their dynamic interactions with tumor and/or stromal cells [52, 53]. According to our literature review, the peritumoral area but not the tumor core itself is the main site for the active infiltration of T cell subsets as CD8+T cells and FoxP3-CD4+ T cells while Tregs infiltrate into the tumors. Therefore, CCA must be considered to be immune-excluded tumors in which most effector T cells are sequestered at the tumor margin [54]. The density of CD8+ T cells in the invasive tumor margin rather than the tumor center demonstrated the best predictive capacity in predicting anti–programmed death (PD)-1 responses in melanoma patients [55]. On the other hand, a higher level of IT but not PT CD8+ T cells in conjunction with certain proteins e.g. PD-1 [23] or CD103 [33] was reported to be associated with outcomes in CCA patients. There are, of course, a few studies [27] with contradicting results, which may be related to different methods, counting standards or sample sizes (Table 2).

Despite the fact that CD20+ B cells make up a small proportion of the total TILs and there is still scarce date on their function, elevated population of B cells have been observed in the lymphoepithelioma-like CCA, a rare subtype of iCCA associated with Epstein-Barr virus (EBV) infection [56]. Huang et al. observed that this lymphoepithelioma-like EBV-associated intrahepatic cholangiocarcinoma (LEL-EBVaICC) subtype had significantly higher densities of CD20+ cells compared with conventional EBVaICC and non-EBVaICC. Additionally, increased density of CD20+ B cells was significantly related to longer OS and RFS in ICC [57]. Thus, one might argue that the LEL subtype of EBVaICC is linked to better prognosis, which might be attributed to local immunological activation by the higher number of tumor-infiltrating B cells and CD8+ T cells. In general, B cells play a variety of roles in the immune system. Tumor-infiltrating B lymphocytes (TIBs) can be observed in various solid tumors. Here, according to existing data, TIBs limit tumor growth by secreting immunoglobulins, boosting T cell response, and directly destroying cancer cells [58]. By supporting the formation and maintenance of tertiary lymphoid structures that promote CTL infiltration into the tumor, TIBs and B cell-related pathways contribute to a powerful anti-tumor response and therefore might improve patient outcomes [59, 60]. Regulatory B cells (Bregs), on the other hand, are thought to facilitate tumor activity by secreting immunosuppressive substances including IL10 and/or TGF-β [61]. Further, it has been shown that B cells can influence tumor growth by interacting with helper T cells [62]. In ovarian cancer, some data suggests that tumors showing both CTLs and B cells have a greater survival probability than tumors containing only CTLs [63]. Two clinical trials in patients with pancreatic ductal adenocarcinoma (PDAC) (NCT02436668) and head and neck cancer (NCT02454179) are currently recruiting to determine the feasibility of utilizing B cells as a potential immunotherapeutic target. However, given the limited amount of quality data on B cells in the general CCA population, further research is warranted to draw valid evidence-based conclusions.

Previous research has shown that distinct CCA subtypes present different risk factors, oncogenic processes and prognoses. Nevertheless, we found no significant changes in TILs between the different CCA subtypes in our present review. CD8+, CD4+, and CD3+ T lymphocytes were mostly peritumoral in both iCCA and eCCA and were found to be favourably linked with overall prognosis. However, the association between Foxp3+ T cells and CCA long-term outcomes is still up for debate. Unfortunately, the prognostic significance of B cells cannot be clearly established due to a lack of studies and additional research in this area is needed in the future.

In the last decade, the molecular pathogenesis (genetic mutations, inflammatory mediators, single pathways, etc) of CCA has been enlightened, and as a result, a number of molecularly targeted therapies (lapatinib, erlotinib, vandetanib, sunitinib, cediranib, ponatinib, etc) have emerged [64]. Nearly 40% of CCA were discovered to have potentially targetable genetic changes such as FGFR2, PRKACA, and ERBB2, implying that targeted molecular therapies could play a role in the clinical management of these patients [65]. Furthermore, tumors with a high mutational burden and a matching upregulation of immune checkpoint markers had the worst prognosis [65]. Therefore, a subset of CCA with specific genetic alterations may provide an opportunity for the combination of small molecule inhibition in combination with immunotherapy [66].

In this systematic review, we found that Wnt/-catenin, TGF-signaling routes, aPKC-i/P-Sp1/Snail Signaling, B7-H1/PD-1Pathway and Fas/FasL signaling pathways were connected to the malignant potential and contributed to tumor immune evasion by increasing TILs apoptosis. In addition, some genes such as *KRAS* and the KIR and HLA loci can also interfere with the immune function of TILs. Complementary, CXCL9, PRKAR1A, IL-10 and TGF-b were associated with the activation of TILs (Table 3). Individualizing treatment choices for patients with advanced CCA may therefore be aided by comprehensive genetic analysis, which holds considerable potential for precision-oncology therapy. The rising problem with immunotherapy appears that only a subgroup of patients benefits from a monotherapy, thus investigating techniques to overcome resistance to immunotherapy should subsequently be a main research area for the future. A significant number of clinical trials will be required to test the combination of molecular targeted treatment and immunotherapy as well as to explore the underlying mechanisms in combined treatments.

**Fig. 4:**
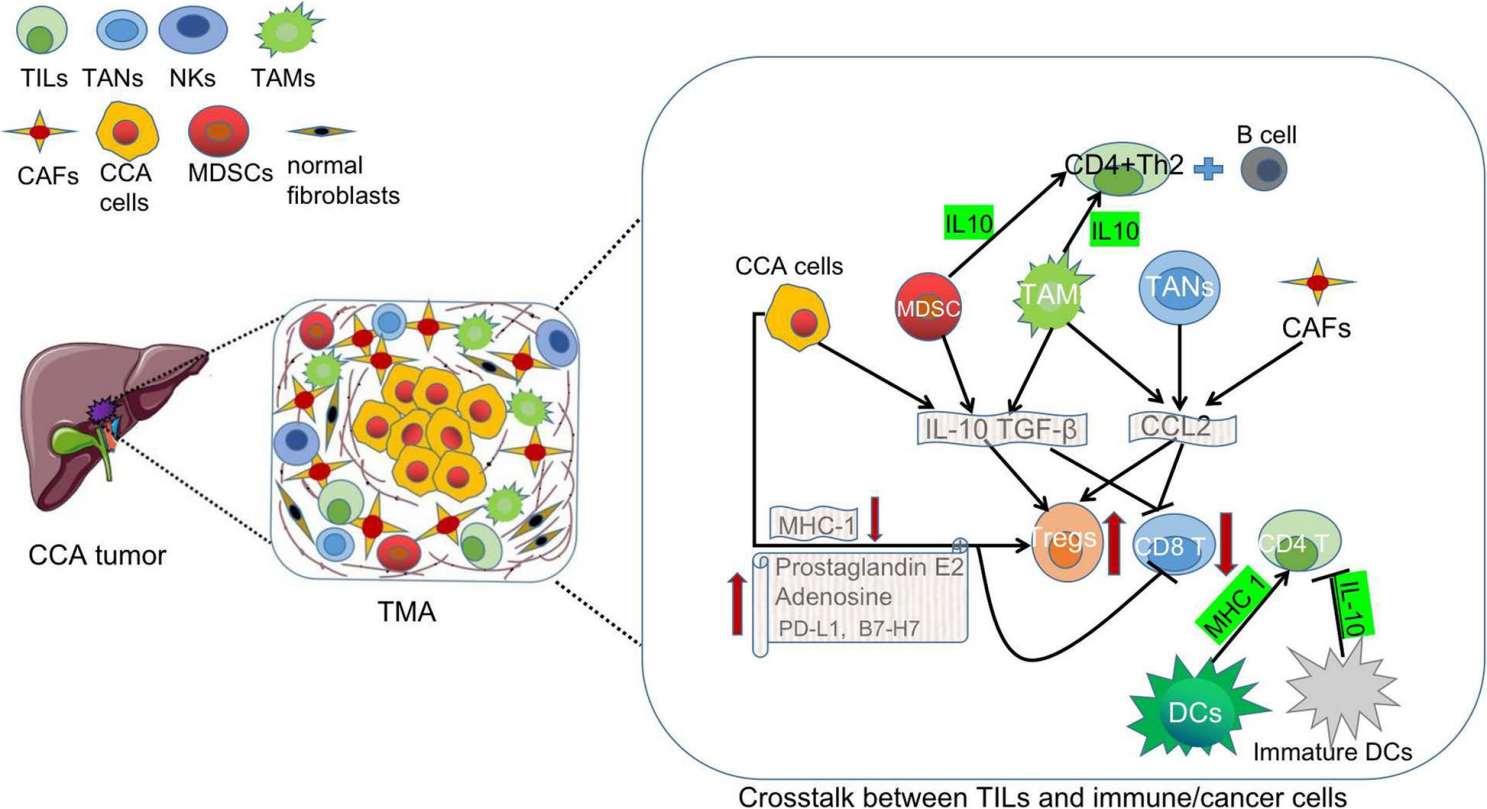
Overview of the crosstalk between TILs and immune/cancer cells in the tumor microenvironment Cancer cells, TAMs and MDSCs emit IL-10 and TGF-β, while TAMs, TANs and CAFs secrete CCL2 which attracts and expands Tregs inside the tumor bed and inhibits the activity of CD8+T cells. Cancer cells can also directly impair the immunoresponse by overexpressing prostaglandin E2, adenosine, PD-L1 or B7-H7 or by lowering MHC-I surface expression. IL10 released by MSDCs and TAMs favors a CD4+ Th2 response with B-cell engagement which are both effective cancer immunosurveillance mechanisms. Mature DCs promote CD4+ T cell activity by increasing MHC 1 expression while immature DCs inhibit CD4+ T activity by secreting IL-10. B7-H7, B7 homolog 7; CAFs, Cancer-associated fibroblasts; CCL,C–C motif chemokine ligand; CD, cluster of differentiation; DCs, Dendritic cells; IL, interleukin; MDSCs, Myeloid-derived suppressor cells; MHC; major histocompatibility complex; PD-L1, Programmed death-ligand 1; TANs, tumor associated neutrophils; TAMs, tumor-accociated macrophages; TGF, transforming growth factor; Tregs, Regulatory T cells

CCA is a desmoplastic cancer with a rich TME where CCA cells exchange autocrine/paracrine signals with each other and other cell types, e.g. cancer-associated fibroblasts (CAFs) and immunosuppressive innate immune cells like tumor-associated macrophages (TAMs) and myeloid-derived suppressor cells (MDSCs). The role of TILs in CCA is therefore strongly affected by the surrounding immune environment as illustrated in Fig. 4. Immune tolerance mechanisms in the tumor microenvironment limit or reduce T-cell responsiveness. Tumor-infiltrating dendritic cells (DCs) with a deficiency in maturation or antigen-presenting cell function have an immunosuppressive or tolerogenic character, inhibiting CD8+ and CD4+ T-cell priming [67, 68]. Furthermore, these DCs commonly contain inhibitory molecules, such as PD-L1 restricting T-cell activation [68]. The failure of the adaptive antitumor immunity is also linked to the polarization of naive CD4+T cells in the tumor microenvironment. Indeed, MDSCs and TAMs emit IL10 and TGF-β [68, 69], while tumor-associated neutrophils (TANs), TAMs, and CAFs secrete CCL2 attracting and expanding the population of Tregs inside the tumor [70–72]. DCs also help to attract Tregs to the tumor and Tregs subsequently support this regulatory phenotype of DCs by expressing the inhibitory immunological checkpoint cytotoxic T-lymphocyte-associated protein 4 (CTLA-4) on a constant basis [67, 68]. Moreover, IL10 released by MSDCs and TAMs favors a CD4+ Th2 response with B-cell engagement over CD4+Th1 and cytotoxic CD8+T (Tc1) responses, which are both effective cancer immunosurveillance mechanisms [69, 73]. These interactions create a *vicious* cycle in which a large number of Tregs generate IL10 and TGF-β attracting more immunosuppressive innate immune cells, which convert dendritic cells into indoleamine 2,3-dioxygenase (IDO)-producing regulatory dendritic cells blocking the immune system from rejecting the cancerous tissue [68, 69].

The capacity of malignant cells to evade immunosurveillance is one of the hallmark features of cancer [74]. Tumor cells, including CCA, recruit protumorigenic immune cells by secreting a variety of immunosuppressive substances such as TGF-β and IL10, which can attract Tregs [70]. By physically blocking T cells, CAFs concentration within CCA malignancies suppresses adaptive antitumor immunity. Production of CXCL12 by CAFs disrupt T-cell migration into tumors [75]. Cancer cells can further directly decrease T-cell-mediated antitumor immunity by overexpressing immune checkpoint ligands like PD-L1, the human endogenous retrovirus-H long terminal repeat-associating protein 2 (also known as B7-H7) [73] or by lowering MHC-I surface expression [76]. CCA cells are also thought to generate prostaglandin E2 and adenosine, both of which can impair T cell function and activity directly [24].

Despite these direct interactions with their microenvironment, TILs might also be involved in lymphovascular invasion, a major feature in cancer progression resulting in lymph node metastases and subsequently reduced survival [77, 78]. Interestingly, a correlation between lymph node metastasis and TILs has been observed in gastric cancer, breast cancer and melanoma [79–81]. In these entities, individuals with high number of TILs were less frequently observed to have nodal metastases. Unfortunately, the underlining mechanisms of these “protective” abilities remain to be elucidated further. Nonetheless, the observation underlines the potential utilization of TILs and TIL-related biomarkers as a prognostic factors in the future [77].

Upcoming research regarding the interaction of TILs with other cells of the TME as well as their sub-characterisation will be highly influenced by the method of single-cell RNA sequencing analysis to elucidate this comprehensive transcriptomic landscape and intercellular communication network. In this context, Zhang et al. recently identified 8 different subtypes of T and NK cells in the TME of iCCA showing different expression of cytotoxic and exhaustion markers [82]. This study particularly underlines the heterogeneity in the fibroblast population (6 distinct subtypes), suggests a high immunosuppressive profile of Tregs and speculates regarding the potential manipulation of Tregs to treat iCCA [82]. One might therefore assume that further reports of single cell approaches in CCA will play an important role in shaping our view on subpopulations and their respective interactions.

Notably, the spatial distribution of immune infiltrates in the tumor microenvironment has been reported to be associated with different clinical implications in patients who received systemic therapy in other cancers [83, 84]. However, little is known about the spatial heterogeneity of immune infiltrates and their clinical implications in CCA. In the analysed literature within this systematic review, different immune cells and their subtypes had different prognostic implications for the long-term outcome in CCA. CD8+T cells show a significant association with prolonged OS irrespective of being detected inside [17, 29] or outside [18] of the tumor. A high density of CD4+ T cells in the tumor margin appeared also to be independently associated with favorable DFS and OS [29]. Additionally, significant numbers of CD20+ cells have been found in low-grade tumors and were linked to a better overall survival [18]. In summary, although there are occasionally conflicting results, CD8+, CD4+, CD3+, CD20+ T and B cells were almost positively correlated with the prognosis of CCA. In contrast, a high number of Tregs is very likely associated with worse OS [28, 38]. Future studies are certainly needed to clarify the prognostic relevance of TIL in long-term outcome in CCA.

Radical and complete surgical resection remains the treatment of choice in all subtypes of CCA in the setting of localized disease. Unfortunately, due to delayed diagnosis and locally advanced situation with the infiltration of adjacent organs or large vessels, most patients do not qualify for curative-intent surgery. Combination chemotherapy with gemcitabine and cisplatin is the current gold standard of care for patients with unresectable or metastatic CCA while several targeted therapy have also been investigated in multiple phase I and II clinical trials [85, 86]. However, the desmoplastic nature of CCA, as well as its broad support from a rich tumor microenvironment and significant genetic variability, contribute largely to resistance to chemotherapy and targeted therapy, resulting in a low overall response rate (ORR) and OS in the palliative setting [87]. TILs play a major role in any immunotherapy approach to CCA, with T cells certainly being the most import part e.g. cytotoxic T lymphocytes recognizing and removing tumor cells, and Tregs having an inhibitory effect. Subsequently, current immunotherapies focus mainly on T cells, such as immune checkpoint (CTLA-4 and PD-1/PD-L) therapies and chimeric antigen receptor (CAR) T cell therapies. However, resistance to immune therapy is still commonly observed in most cancer patients [88]. As a result, finding strategies to aid and boost immunotherapy is critical. For instance, Interleukin 2 (IL-2) plays an important role in the immune system and participates in the signal transduction of T cells which has now been widely investigated [89]. While improved IL-2 formulations may be used as monotherapies, their combination with other anticancer immunotherapies, such as adoptive cell transfer regimens, antigen-specific vaccination, and blockade of immune checkpoint inhibitory molecules, e.g. cytotoxic T lymphocyte-associated antigen 4 (CTLA-4) and programmed death 1 (PD-1) mono-antibodies, have the potential to treat metastatic cancer [90]. Research has shown that anticancer drugs that are rationally selected for triggering tumor immunogenicity can be used to make resistant tumors sensitive to checkpoint blockade therapy [91, 92]. Anticancer drugs promoting apoptosis are thought to be the most effective at boosting the immune system. Antigens generated by dying tumor cells have been demonstrated to be potent immune stimulators when delivered through antigen-presenting dendritic cells. However, chemotherapy is of course historically known to be itself also immunosuppressive. Spreafico et al. demonstrated that daunorubicin had rather good anticancer cytotoxic action but was also immunosuppressive. On the other hand, the hydroxylated congener of doxorubicin demonstrated potent anticancer efficacy while causing minimal immunosuppression. When daunorubicin was used against a tumor that was highly susceptible to its cytotoxic effects, mice were cured regardless of immunosuppression, but when the tumor was reasonably resistant, the immunosuppressive effects were significantly more visible and predominant [93]. We reviewed the possible methods of assisting TIL immunity in the past 20 years, such as anti-CD40, CXCL9, cytokine-activated killer (CAK), and gemcitabine (Table 5). These substances directly or indirectly affected the immune function of TIL, thereby promoting the immunotherapy of CCA. Although these novel ways are primarily based on in vitro studies and animal models, they provide the theoretical foundation for future clinical translation.

Clinical evidence on immune-directed treatments in CCA is still limited. Immunotherapy methods such as immune checkpoint inhibitors have been investigated in CCA but did not display a very convincing effect [94]. While these studies mostly investigated the palliative setting, it might be possible that particular in the neoadjuvant setting, more pronounced effects might be observed as the initial tumor has a greater endogenous tumor antigen load which might improve T-cell priming by immunotherapy and facilitate the eradication of micrometastases leading to disease recurrence after surgery [94, 95]. Therefore, numerous ongoing trials in CCA are evaluating the safety and effectiveness of ICI in the neoadjuvant (NCT03768531) or adjuvant setting (NCT03820310) in the surgical scenario. These results are eagerly expected. PD-L1 and CD3 expression [96] in tumor tissue, inflammatory signatures such as INF-γ-related mRNA proflie [96], and T cell exhaustion signature [97] have all been found to be linked to increased survival and therapy response in patients with advanced HCC treated with single-agent anti-PD-1 treatment. However, more studies on TILs in CCA are required to unravel the potential of TIL-related biomarkers to predict immunotherapy response in this scenario.

This review has certainly one major limitation which have to be discussed critically. Due to the low number of studies including oncological outcome and the heterogeneous reporting standards, we were not able to conduct a reasonable meta-analysis of the prognostic effects of TILs in CCA. Further studies contributing to the understanding oncological role of TILs should therefore be a main focus of research in CCA.

## Conclusion

The aim of this systematic review was to examine the current literature available on the proportions and distribution, molecular pathogenesis, prognostic significance and potential immunotherapy TILs in CCA patients. The hereby summarized literature suggest that TILs may represent an important marker for the prognosis of the CCA. Further, TILs play a major role in immunotherapy for CCA, but more clinical data is needed to fully explore the importance of TIL in the context of novel clinical treatments.

## Data Availability

Available upon request

## Abbreviations

ACT: Adoptive cell therapy
aPKC-i: Atypical protein kinase C-iota
B7-H7: B7 homolog 7
BTC: Biliary tree cancers
CAFs: Cancer-associated fibroblasts
CAK: Cytokine-activated killer
CAR: Chimeric antigen receptor
CCA: Cholangiocarcinoma
CCL2: C–C motif chemokine ligand 2
CD: Cluster of differenciation
c-FLIP: Cellular FADD-like IL-1β-converting enzyme-inhibitory protein
CI: Confidence interval
CTLs: Cytotoxic T-lymphocytes
CTLA-4: Cytotoxic T-lymphocyte antigen-4
CXCL9: Chemokine (C-X-C motif) ligand 9
DCs: Dendritic cells
dCCA: Distal cholangiocarcinoma
FoxP3: Forkhead box P3
DFS: Disease free survival
FasL: Fas ligand
HBV: Hepatitis B virus
HLA: Human leukocyte antigen
HR: Hazard ratio
iCCA: Intrahepatic cholangiocarcinoma
IDO: indoleamine 2,3-dioxygenase
IHC: Immunohistochemistry
IL: Interleukin
INF: Interferon
IT: Intratumor
KIR: Killer cell immunoglobulin-like receptor
MDSCs: Myeloid-derived suppressor cells
MHC II: Major histocompatibility complex II
MAKP-ERK: Mitogen-activated protein kinases-Extracellular signal-regulated kinases
mRNA: Messenger RNA
MSI-H: Microsatellite instability
NK: Natural killer
OS: Overall survival
ORR: Overall response rate
PRKAR1A: Protein Kinase CAMP-Dependent Type I Regulatory Subunit Alpha
pCCA: Perihilar cholangiocarcinoma
PCR: Polymerase chain reaction
PD-1: Programmed death-1
PDAC: Pancreatic ductal adenocarcinoma
PD-L1: Programmed death-ligand 1
PFS: Progression-free survival
PRISMA: Preferred Reporting Items for Systematic Reviews and Meta-analyses
P-Sp1: Ser59-phosphory-lated specificity protein 1
PT: Peritumor
RRS: Recurrence rate
siRNA: Small interfering RNA
TAMs: Tumor-associated macrophages
TANs: Tumor-associated neutrophils
TIBs: Tumor-infiltrating B lymphocytes
TIL: Tumor-infiltrating lymphocyte
TGF: Transforming growth factor
TM: Tumor margin
Tregs: Regulatory T cells
Wnt: Wingless and Int-1
